# Association of reading problems with speech and motor development: results from a British 1946 birth cohort

**DOI:** 10.1111/j.1469-8749.2010.03649.x

**Published:** 2010-03-19

**Authors:** Darya Gaysina, Barbara Maughan, Marcus Richards

**Affiliations:** 1 MRC Unit for Lifelong Health and Ageing, University College LondonLondon, UK; 2 MRC Social, Genetic and Developmental Psychiatry Centre, King’s College London, Institute of PsychiatryUK

SIR–We investigated, using data from the Medical Research Council (MRC) National Survey of Health and Development (the British 1946 birth cohort; *n*≥3083), whether later attainment of developmental milestones and speech problems (stammering or articulation problem) were associated with reading problems at age 11 years, and whether children with reading difficulty were at increased risk of motor problems in adolescence. Multiple linear regression and logistic regression were applied for the analyses. The results showed that reading problems were associated with later age at standing (*p*=0.006) and walking (*p*=0.048). Speech problems (articulation problems and stammering) at age 6 and 7 years were associated with reading difficulty. In contrast, we did not find any association between reading problems and fine motor skills at age 15 years. Our study concludes that delayed gross motor development and early evidence of speech problems predict risk of reading impairment; however, reading impairment may not be associated with motor problems.

Co-occurrence of reading difficulties and motor coordination problems has been reported in several studies.^1^–^3^ Speech problems are also associated with reading difficulties.^4^–^7^ Mechanisms underlying overlapping problems in domains of reading, speech, and motor function remain poorly characterized. One hypothesis is that these associations reflect common neural substrates for different motor and cognitive functions at varying developmental stages. The neurodevelopmental hypothesis is supported by the observation that developmental milestones may be delayed in children with reading difficulties,^8^–^10^ although relevant studies to date are based on relatively small samples and/or specific samples, and are not well controlled for potential confounders.

Using longitudinal data from the British 1946 birth cohort, we tested whether later attainment of gross motor developmental milestones and early speech problems can predict reading difficulties in childhood, and whether children with reading problems are more likely to experience problems with speech and motor control skills in adolescence.

At age 11 years, 4307 survey members were contacted, and 4281 (99.4%) were administered tests of general cognitive ability and reading ability by teachers in a school setting. The general ability test consisted of 80 verbal and non-verbal reasoning items, shown to have satisfactory reliability and validity.^11^ The total score was normalized to a mean of 100 and a standard deviation (SD) of 15 to give a score comparable to IQ. At the same age survey members were also asked to read aloud 50 words, ranging from low difficulty (e.g. egg, book) to high difficulty (e.g. extraneous, ophthalmic). Children were classified as having reading problems if they achieved a general ability score ≥70, and a reading score <20.68 (1.5 SD below the mean; *n*=312). The group scoring at or above the threshold on both tests provided a comparison group (*n*=3294).

When cohort children were 2 years old their mothers were asked about age at sitting, standing and walking unaided, and age at saying words other than names for parents. At age 6, 7, and 15 years a medical examination by a physician at school recorded if the child had any speech difficulties (stammering or articulation problem). At age 15 years a medical examination by a physician at school recorded, with the study member seated, speed of tapping the dorsum of the right hand with the left finger (and vice versa) and tapping the ground with the right and then left foot.

Associations between reading problems (yes/no) and developmental milestones, as well as between reading problems and motor or speech difficulties, were tested using regression models. Potential confounders were sex, birth rank (first child or not), father’s occupational class (manual/non-manual), parents’ education (primary/secondary), material home conditions (occupation per room), and non-verbal intelligence score at age 11 years.

Later ages at standing and walking were associated with increased risk of reading problems at age 11 years after controlling for all confounders (Table I). There were no significant associations between ages at sitting or speech and reading problems. Speech problems at age 6 or 7 years were significantly associated with reading problems. There were no associations between reading problems and motor skills, and speech problems at age 15 years

**Table I:** Associations between reading problems (yes/no) and age at developmental milestones attainment, speech, and motor functions: regression coefficients presented for a single predictor, effects adjusted for early background and non-verbal intelligence at age 11 years

		Associations with reading problems		
		
	*n*	Coef.	(95% CI)	*p* value
Predictors^a^				
Age at sitting	3302	0.06	(−0.03; 0.16)	0.20
Age at standing	3302	0.09	(0.03; 0.15)	0.006
Age at walking	3302	0.05	(0.00; 0.11)	0.048
Age at speech	3106	0.01	(−0.03; 0.04)	0.69
Stammering at age 6–7y	3328	1.11	(0.10; 2.13)	0.03
Articulation problem at age 6–7y	3328	0.99	(0.53; 1.46)	<0.0001
Outcomes^b^				
Finger-tapping at age 15y	3083	−1.11	(−3.65; 1.43)	0.39
Foot-tapping at age 15y	3083	−1.19	(−3.63; 1.25)	0.34
Stammering at age 15y	3182	0.99	(0.05; 1.94)	0.04
Articulation problem at 15y	3182	0.40	(−0.87; 1.67)	0.54


a In these regression models, reading problems (yes/no) is the dependent variable, while age at developmental milestones attainment, and stammering and articulation problems are single predictors; ^b^In these regression models, motor (finger-tapping, foot-tapping) or speech (stammering or articulation problem) functions are the dependent variables, while reading problems (yes/no) is a single predictor. Coef, coefficient; CI, confidence interval.

Our findings for delayed motor development in children with reading problems are in agreement with some previous studies,^8^, ^9^, ^12^ and extend the results to the general population. We also found that the relationship between child development and reading problems extends to speech development, which is consistent with previous findings.^6^, ^7^ Our study has several strengths, including a large representative sample of children of the same age, maternal reports of motor development collected close to the events, and information on confounders collected prospectively.

Despite evidence of comorbidity between reading disability and motor and speech impairment the field has yet to reach a consensus about the precise cause of this association. It has been shown that the cerebellum plays an important role in reading difficulty.^13^ The cerebellum also contributes to speech motor control.^14^ One hypothesis states that cerebellar involvement in cognitive modulation may be established through the cerebello-cerebral network.^15^ Thus, the problems within reading, speech, and motor domains could have a common biological substrate operating through the cerebellum.

In conclusion, this study provides further support for the applicability of delay in early developmental milestones as an additional indicator of risk for reading problems.

**What this study adds**

- Delayed ages at standing and walking predict risk of reading impairment in the general population.
- Children with reading difficulty at age 11 years have early evidence of speech problems.
- Reading impairment may not be associated with motor problems in adolescence.
